# Exploring the gut microbiota of Pacific white shrimp (*Litopenaeus vannamei*) suffering pale shrimp disease

**DOI:** 10.1371/journal.pone.0336700

**Published:** 2025-11-11

**Authors:** Lalitphan Kitsanayanyong, Natnicha Chongprachavat, Tirawat Rairat, Arunothai Keetanon, Parattagorn Wimanhaemin, Niti Chuchird

**Affiliations:** 1 Department of Fishery Products, Faculty of Fisheries, Kasetsart University, Bangkok, Thailand; 2 Department of Fishery Biology, Faculty of Fisheries, Kasetsart University, Bangkok, Thailand; Universidad Nacional Autonoma de Mexico Instituto de Biotecnologia, MEXICO

## Abstract

Pale shrimp disease is an emerging threat in Thailand, characterized by pale body coloration in Pacific white shrimp (*Litopenaeus vannamei*). Although the etiology had been identified as *Photobacterium damselae* subsp. *damselae*, the disease effects on gut microbiome remain poorly understood. This study investigated changes in the gut microbiota of Pacific white shrimp suffering from pale shrimp disease (diseased group) compared to disease-free shrimp (healthy group) collected from Surat Thani Province, Thailand. DNA extracted from the intestinal samples was subjected to *16S rRNA* metagenomic sequencing, followed by taxonomic identification, diversity analyses, and functional prediction of the metabolic pathways. Despite a limited number of biological replicates, the occurrence of pale shrimp disease was able to reveal alterations in intestinal microbial composition, diversities, and functional features compared to the healthy shrimp. In most cases, the intestinal microbiota of the diseased shrimp were dominated by only 2 genera of bacteria, i.e., *Photobacterium* (54.63–70.53%) and *Vibrio* (24.94–26.12%), which together accounted for 79.58–95.47% of the total bacterial community. α-diversity, as indicated by the observed features, Shannon, and Simpson indices, was significantly decreased, and dominance was significantly increased in the diseased shrimp compared to healthy shrimp. Likewise, β-diversity was significantly different between groups; PCoA of un-weighted and weighted UniFrac clearly distinguished intestinal microbiota of the shrimp into 2 clusters, and ANOSIM of these data revealed statistical differences between groups, suggesting different microbiota communities between healthy and diseased shrimp. Moreover, diseased shrimp had significantly higher predicted functional features associated with bacterial virulence factors and antibacterial resistance. These exploratory findings suggest an association among pale shrimp disease, gut microbiota dysbiosis, and the proliferation of opportunistic taxa, particularly *Photobacterium*.

## Introduction

Pale shrimp disease, which is characterized by uniformly pale body coloration, is an emerging disease of Pacific white shrimp (*Litopenaeus vannamei*) in Thailand. The outbreaks have been reported from commercial shrimp farms that use low-salinity systems in Surat Thani Province, Southern Thailand since 2022. Later, the causative agent was determined as *Photobacterium damselae* subsp. *damselae* (PDD) [1]. Although the etiology of pale shrimp disease has been identified, our understanding of the disease occurrence and pathogenesis is still at an early stage.

Gut microbiota play an important role in promoting the host’s health; they modulate nutrient absorption, immune responses, and disease resistance [2,3]. Gut microbiota composition of aquatic animals can be influenced by diet components, feed additives, antibiotic treatments, environmental conditions (such as water salinity and temperature), developmental stage, and health status [4–6]. Regardless of these factors, Proteobacteria is by far the most dominant phylum in the intestines of Pacific white shrimp, followed by phyla Bacteroidetes, Actinobacteria, and Firmicutes [2,5].

The bacterial community structure of healthy shrimp is typically distinct from that of diseased one. For example, Pacific white shrimp suffering from white feces syndrome (WFS) [7–9], acute hepatopancreatic necrosis disease (AHPND) [10–12], translucent post-larvae disease (TPD) [13], and nitrite stress [14] usually contain more *Vibrio* but less *Candidatus* Bacilloplasma in their intestines compared to healthy shrimp. Furthermore, higher levels of *Photobacterium* have been observed in the intestine or stomach of shrimp suffering from AHPND [11,12], white spot syndrome virus (WSSV) [15], and environmental stressors such as nitrite [14], ammonia, and sulfide [16]. The stomach of AHPND-affected shrimp under low salinity stress (10 ppt) also contains more *Photobacterium* compared to that reared in optimal salinity water (20 ppt) [17]. However, reports on *Photobacterium* abundance in WFS-affected shrimp were less consistent [8,18]. It has been suggested that alterations in microbiome composition (dysbiosis) may be responsible for the disease development, making them potential indicators of shrimp diseases [19,20].

Unsurprisingly, as a new emerging disease, studies on shifts in the gut microbial community in the pale shrimp disease have never been conducted. To the best of the authors’ knowledge, gut microbiome investigation in clinically similar diseases, characterized by idiosyncratic white muscle, has been performed in only one instance, called “cotton shrimp-like” disease. The authors found that bacteria of the family Rickettsiaceae and genus *Tenacibaculum* were significantly more abundant in the intestine of diseased shrimp, but their contributions to the disease pathogenesis remain unclear [21]. Since *Photobacterium* was not reported in the cases of cotton shrimp-like disease, it is highly unlikely that this condition is the same as pale shrimp disease. Thus, the changes in gut microbiota in the case of pale shrimp disease remain to be explored.

The current study aimed to investigate how the pale shrimp disease affected the gut microbiota of Pacific white shrimp. *16S rRNA* metagenomic sequencing followed by taxonomic identification, diversity analyses, and functional prediction of the metabolic pathways, were employed. During the occurrence of pale shrimp disease, a reduction in the intestinal microbiota and diversity was expected. Furthermore, pathways associated with several virulent factors should be enhanced in the shrimp suffering pale shrimp disease.

## Materials and methods

### Shrimp sample collection

The diseased shrimp samples from the outbreak pond (9–12 g, 91 days old after stocking in the grow-out pond) were collected from 5 different locations of an earthen pond using a low-salinity system (10 ppt) in Surat Thani Province, Thailand in March 2024. Note that this farm has persistently been affected by pale shrimp disease, in which the etiology was identified as PDD in our recent study [1]. Starting from 55 days after stocking, approximately 5–10% of the total shrimp population in the outbreak pond exhibited a characteristic clinical sign of pale body coloration. Some affected shrimp also showed gross signs of pale hepatopancreas and empty intestine, indicating poor health conditions. Realizing that the disease might not be easily controlled, the farmer finally decided to terminate the grow-out phase on day 91 after stocking with a mortality rate of 70%. In addition, apparently healthy shrimp of similar size and age from a disease-free aquaculture farm nearby (without disease outbreak) were also collected as a control group (healthy shrimp). Entry and sample collection were approved by the farm owners. The husbandry practices were identical between the healthy and diseased ponds. Post-larvae of both groups were obtained from a single commercial hatchery, stocked at the same density of 75 shrimp/m^2^, and were fed the same commercial feed (containing 40% protein, 4% fat, and 4% fiber). Water quality parameters during culture were maintained in an appropriate range for shrimp culture: dissolved oxygen was > 5 mg/L; pH was 7.8–8.3; salinity was 10 ppt; alkalinity was 120–150 mg/L as CaCO_3_; total ammonia was < 1 mg/L; and nitrite was < 0.5 mg/L.

The live shrimp samples were transported under aeration, with the water temperature kept below 28 °C. Upon arrival at the Aquaculture Business Research Center (ABRC) laboratory, Faculty of Fisheries, Kasetsart University, Bangkok, Thailand, shrimp were subjected to disease screening test, bacterial isolation and reinoculation, and microbiome analysis. The shrimp were humanely euthanized using a 2-step approach in accordance with the AVMA Guidelines [22]. They were immersed in 5% ethanol for 10 min to induce anesthesia, followed by pithing to ensure death. The animal study was approved by the Kasetsart University Institutional Animal Care and Use Committee (IACUC approval number: ACKU66-FIS-018).

### PCR detection of major shrimp pathogens

The shrimp from both healthy and diseased ponds were screened for some important shrimp diseases including AHPND, WSSV, infectious myonecrosis virus (IMNV), yellow head virus (YHV), and decapod iridescent virus 1 (DIV1) using PCR techniques following the published protocols [1]. None of these pathogens were detected in the healthy and diseased ponds.

### Bacterial isolation and infection challenge

Hepatopancreas from diseased shrimp were collected, homogenized, and streaked on thiosulfate-citrate-bile salts-sucrose (TCBS) agar and tryptic soy agar (TSA) supplemented with 1.5% NaCl. After incubation at 37 °C overnight, representative single colonies on each agar were randomly selected and subjected to bacterial identification through *16S rRNA* sequencing and BLAST homology search following the published protocols [1]. *Vibrio parahaemolyticus*, *V. vulnificus*, *V. alginolyticus*, *V. mimicus*, and *Photobacterium damselae* were identified. Subsequently, individual isolate was reinoculated via oral gavage into the healthy shrimp (10^7^ CFU/shrimp) following our previous study’s protocols [1]. Out of several bacterial isolates, only *P. damselae* could cause pale shrimp body.

### DNA extraction

Five pooled intestinal samples of healthy shrimp (n = 5) and diseased shrimp (n = 5) were preserved in NAPseq^™^ Nucleic Acid Preservation Buffer (BioEntist Co., Ltd., Thailand) and sent to Omics Sciences and Bioinformatics Center (Chulalongkorn University, Bangkok, Thailand) for DNA extraction and sequencing. Each pooled sample consisted of intestines from 5 individual shrimp. DNA in the intestinal sample of healthy and diseased shrimp was extracted using DNeasy PowerSoil Pro DNA Kit (Qiagen, USA) following the protocol described by the manufacturer. The quality and quantity of the extracted DNA were assessed by a Nanophotometer N50 (Implen, Germany) while the integrity was determined on 1.2% agarose gel electrophoresis.

### *16S rRNA* metagenomic sequencing

The V3-V4 variable region of the bacterial *16S rRNA* was amplified by PCR in 25-µL reaction volume using 100 ng DNA, 2X sparQ HiFi PCR master Mix (QuantaBio, USA), and 0.2 µM of each forward and reverse primer with overhanging adaptors. Details of the primers’ sequences are as follows: 341F (5′-TCGTCGGCAGCGTCAGATGTGTATAAGAGACAGCCTACGGGNGGCWGCAG-3′) and 805R (5′- GTCTCGTGGGCTCGGAGATGTGTATAAGAGACAGGACTACHVGGGTATCTAATCC- 3′) where the underlined sequence are overhanging adaptors, and the rest are complementary sequences to V3-V4 region of the bacterial *16S rRNA*. The PCR amplification was initiated by initial denaturation at 98 °C for 2 min, followed by 30 cycles of denaturation at 98 °C for 20 s, annealing at 60 °C for 30 s, and extension at 72 °C for 1 min, then final extension at 72 °C for 1 min. The resulting PCR amplicons of approximately 550 bp were checked by 1.2% agarose gel electrophoresis with RedSafe (iNtRON Biotechnology, Inc., Korea) staining, purified by sparQ Puremag Beads (QuantaBio, MA, USA), and diluted to 50 µL. A 5-µL volume of the PCR products was indexed with 2.5 μL of each of the forward and reverse Nextera XT index primer (Illumina, CA, USA) in a 50-μL reaction, and was amplified again following the amplification condition described above for 10 cycles. Then, the final *16S rRNA* amplicons of approximately 630 bp were cleaned, pooled, diluted to 4 pM, and loaded onto an Illumina MiSeq (Illumina, CA, USA) where the clusters were generated and 250-bp paired-end read sequencing was performed. Sequencing data have been deposited in the NCBI SRA under BioProject accession PRJNA1328360 with associated BioSamples SAMN51331860–SAMN51331869 and SRA accessions SRR35391129–SRR35391138.

### Bioinformatic and statistical analysis

Data processing and microbiome bioinformatic analysis were performed using Quantitative Insights Into Microbial Ecology 2 (QIIME 2) version 2023.9 [23]. q2‐demux plugin was used to demultiplex the obtained sequencing data. Low-quality reads were removed, and data were denoised by q2-dada2 plugin [24]. Amplicon sequence variants (ASVs) of less than 18 reads, corresponded to microbiome errors in this study, and the reads of mitochondria and chloroplast were filtered out. SATé-enabled phylogenetic placement (SEPP) q2-plugin was used to build a phylogenetic tree [25]. Samples were normalized to the same sequencing depth at 75,219, which correlated to the minimum sequencing number of all the samples.

Taxonomic classification of each ASV was assigned by q2‐feature‐classifier [26] classify‐sklearn naïve Bayes taxonomy classifier using the Silva 138 database based on 99% identity and visualized by bar plot creating by R version 4.4.2 (R Foundation for Statistical Computing, Austria) and heatmap. Venn diagram was built to clarify the number of shared and unique ASVs between healthy and diseased shrimp. Indices of α‐diversity (observed features, dominance, Shannon, and Simpson) and β-diversity, visualized by multidimensional principal coordinate analysis (PCoA) of un-weighted and weighted UniFrac distance [27] were estimated using q2‐diversity. R program was used to create boxplot for visualization of α‐diversity indices. Kruskal-Wallis, and Permutational Multivariate Analysis of Variance (PERMANOVA) and Analysis of Similarity (ANOSIM) at P < 0.05 were used to identify differences in the α‐ and β-diversity between groups, respectively.

The potent functional features of intestinal microbiota were annotated based on *16S rRNA* sequences using Phylogenetic Investigation of Communities by Reconstruction of Unobserved States 2 (PICRUSt2) and the Kyoto Encyclopedia of Genes and Genomes (KEGG) database at level 1, 2, and 3 KEGG ortholog groups (KOs) [28]. STAMP software [29] was used to compare the differential abundance of predicted functional features between groups and illustrated as extended error bar plots. Significant differences (P < 0.05) were analyzed by Fisher’s exact-test with Newcombe-Wilson CI method and Benjamini-Hochberg FDR correction.

## Results

### Taxonomic classification of intestinal microbiota in healthy and diseased shrimp

**Fig 1:**
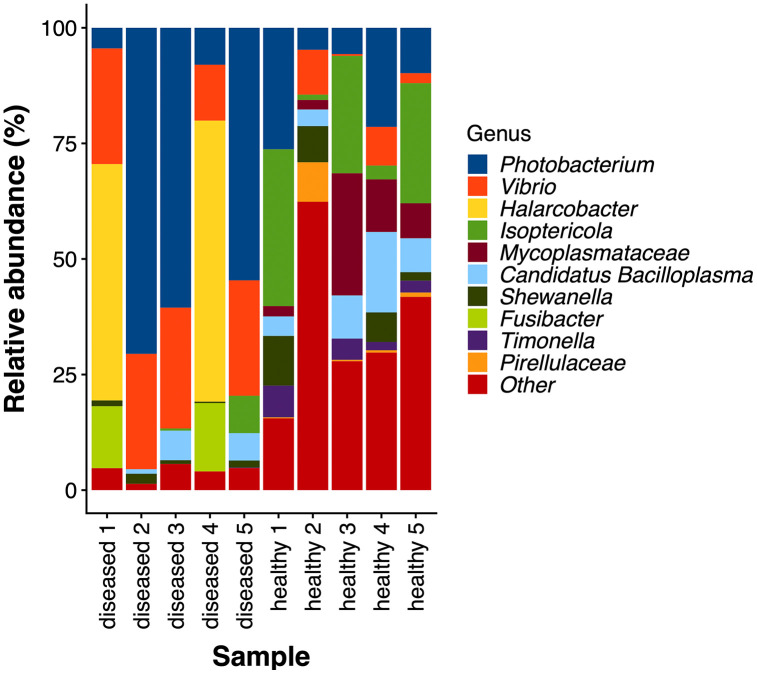
Taxa bar plot of intestinal microbiota from healthy and diseased shrimp at the genus level. The relative abundance of each intestinal microbiota is shown. Each bar represents the data of a pooled intestinal sample in each group (n = 5).

**Fig 2:**
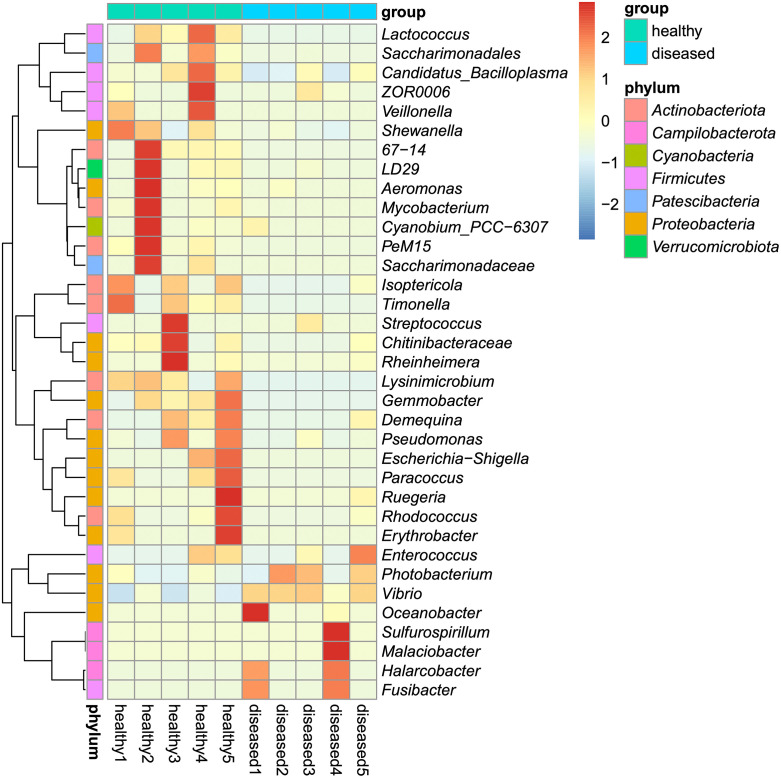
Heat map of intestinal microbiota from healthy and diseased shrimp at the genus level. Each column represents the data of a pooled intestinal sample in each group (n = 5). Color indicated on the map represents the relative abundance of each intestinal microbiota: Red coloration represents higher relative abundance, while blue coloration represents lower relative abundance. Clustering analysis is shown as tree on the left side of the map.

There were 75,219 high-quality reads after data processing, grouped into 385 ASVs, assigned to 15 phyla, 30 classes, 74 orders, 107 families, and 147 genera. The relative abundances (%) of each taxon are illustrated in Fig 1 and S1–S3 Figs. The top 3 dominate phyla in healthy shrimp were Proteobacteria (23.99–43.39%), Actinobacteriota (14.48–47.38%), and Firmicutes (8.24–37.62), while those of the diseased shrimp were Phyla Proteobacteria (20.84–98.29%), Campilobacterota (0–64.15%), and Firmicutes (0.99–4.84%). Within those phyla, Gammaproteobacteria (21.90–40.01%), Actinobacteria (13.00–46.73%), and Bacilli (7–37.61%) were dominant classes of microbiota in healthy shrimp while those in diseased shrimp were Gammaproteobacteria (20.84–98.24), Campylobacteria (0–64.15%), and Clostridia (0–14.79%), respectively. Most of the class Gammaproteobacteria in both healthy and diseased shrimp was family Vibrionaceae (6.05–29.79% and 20.07–95.47%, respectively). Family Promicromonosporaceae (1.16–33.92%) and Mycoplasmataceae (5.66–33.75%) dominated class Actinobacteria and Bacilli in healthy shrimp, respectively, while family Arcobacteriaceae (0–62.39%) and Fusibacteriaceae (0–14.97%) dominated class Campylobacteria and Clostridia in diseased shrimp, respectively. At the genus level, the intestinal microbiota of the shrimp from the diseased pond were dominated by a few bacterial genera and could be clearly separated into 2 types, namely, the *Photobacterium*-*Vibrio* dominant type (3/5 samples) and the *Halarcobacter*-*Fusibacter* dominant type (2/5 samples). In the first group, the abundance of *Photobacterium* (ranging from 54.63–70.53%) and *Vibrio* (ranging from 24.94–26.12%) collectively accounted for 79.58–95.47% of the total bacterial community, while neither *Halarcobacter* nor *Fusibacter* was detected. In the second group, the abundance of *Halarcobacter* (ranging from 51.14–60.80%) and *Fusibacter* (ranging from 13.41–14.78%) comprised 64.55–75.59% of the gut microbial community, whereas those of *Photobacterium* and *Vibrio* were only 4.46–7.99% and 12.08–25.01%, respectively. On the contrary, the intestinal bacterial community of the healthy shrimp was more diverse. The important bacteria include *Isoptericola* (ranging from 1.16–33.92%), Mycoplasmataceae (uncultured) (ranging from 1.51–33.81%), *Candidatus* Bacilloplasma (ranging from 4.22–17.41%), *Shewanella* (ranging from 0–10.76%), *Vibrio* (ranging from 0–9.69%), and *Timonella* (ranging from 0–6.84%). Moreover, a heatmap of microbial genera was constructed to illustrate the differences in the intestinal microbial composition of healthy and diseased shrimp (Fig 2). The results clearly showed the higher microbial abundance in each group. Genera *Lactococcus*, *Saccharimonadales*, *Candidatus* Bacilloplasma, *Shewanella, Isoptericola*, *Timonella*, *Streptococcus*, *Chitinibacteraceae*, *Rheinheimera*, *Lysinimicrobium*, *Gemmobacter*, *Demequina*, *Pseudomonas*, *Escherichia-Shigella*, *Paracoccus*, *Rhodococcus*, *Erythrobacter*, and *Enterococcus* were observed in the higher abundance in healthy shrimp. On the other hand, genera *Photobacterium*, *Vibrio*, *Halacobactor*, and *Fusibacter* were elevated in diseased shrimp, while those of the healthy shrimp were much less.

**Fig 3:**
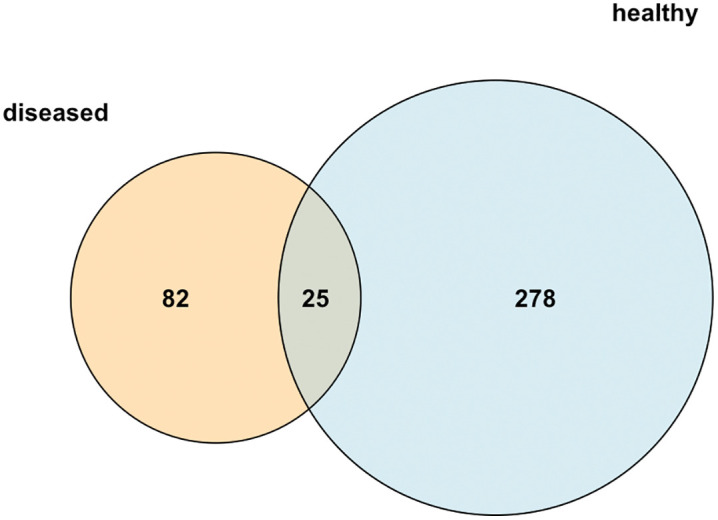
Venn diagram based on ASVs of intestinal microbiota from healthy and diseased shrimp. Number on blue and orange coloration circles represent unique ASVs of intestinal microbiota in healthy and diseased shrimp, respectively, while number on the overlap circle represents shared ASVs of intestinal microbiota in healthy and diseased shrimp. Data were generated based on 5 pooled intestinal samples from each group (n = 5).

Of the 385 ASVs found, shared and unique ASVs in the intestine of healthy and diseased shrimp are shown in Fig 3. There were 25 share and 278 and 82 unique ASVs in intestine of healthy and diseased shrimp, respectively. The lists of share and unique microbiota are shown in S1–S3 Tables. The shared genera were *Vibrio*, *Photobacterium, Isoptericola*, *Candidatus* Bacilloplasma, *Aeromonas*, *Timonella*, *Demequina, Pseudomonas, Blastopirellula*, and unknown genus in the family Caldilineaceae. It should be noted that even though the genus *Photobacterium* was observed in both healthy (7 ASVs) and diseased (10 ASVs) shrimp, there were 3 unique *Photobacterium* ASVs in the diseased group, but no in the healthy shrimp (S1–S3 Tables).

### α-diversity of intestinal microbiota in healthy and diseased shrimp

**Fig 4:**
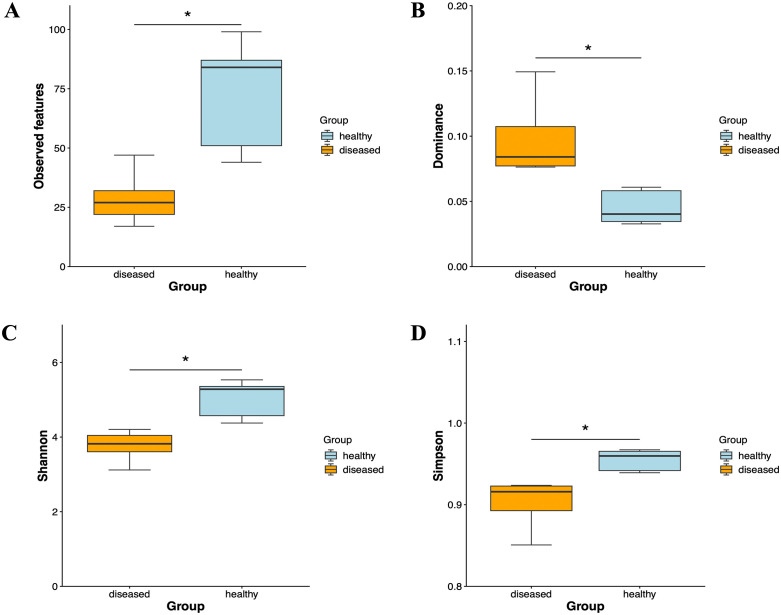
α-diversity indices of intestinal microbiota from healthy and diseased shrimp. (**A**) Observed features, (**B**) Dominance, (**C**) Shannon, and (**D**) Simpson. Data were generated based on 5 pooled intestinal samples from each group (n = 5).

α-diversity is generally used to determine the diversity within the sample [30]. In this study, the observed features, Shannon, and Simpson indices were significantly reduced in the diseased shrimp compared to the healthy shrimp (P < 0.05), and the dominance was significantly increased (P < 0.05) (Fig 4). These results indicated that pale shrimp disease caused reductions in α-diversity.

### β-diversity of intestinal microbiota in healthy and diseased shrimp

**Fig 5:**
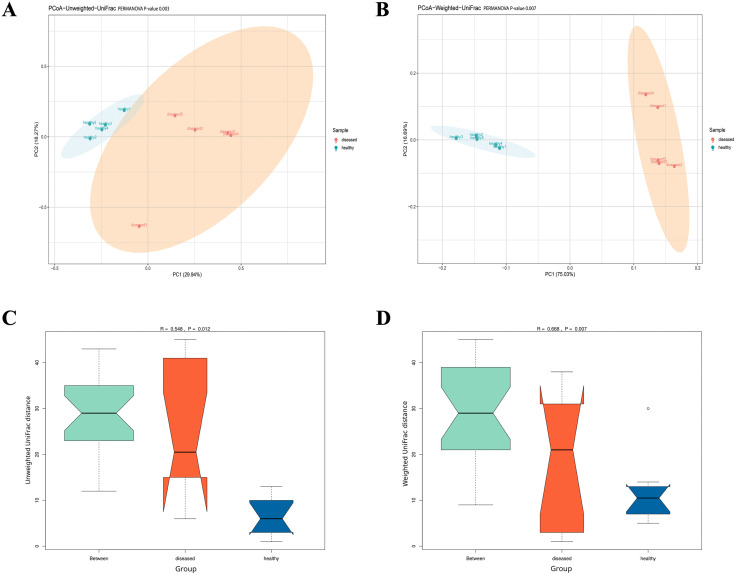
β-diversity of intestinal microbiota from healthy and diseased shrimp. PCoA of (**A**) un-weighted and (**B**) weighted UniFrac distance with PERMANOVA P-value. Boxplot of ANOSIM based on (**C**) un-weighted and (**D**) weighted UniFrac distance with P- and R-value. Data were generated based on 5 pooled intestinal samples from each group (n = 5).

Principal co-ordinate analysis (PCoA) based on the un-weighted and weighted UniFrac distances was conducted to determine the differences in the β-diversity of microbiota in the intestinal of healthy and diseased shrimp (Fig 5A and 5B). Additionally, the PERMANOVA (Fig 5A and 5B) and ANOSIM (Fig 5C and 5D) tests were conducted to conﬁrmed statistically differences of microbial community between groups. The results showed that the intestinal microbiota of healthy and diseased shrimp was clearly separated and clustered into different groups, with PERMANOVA possessing P = 0.003 and 0.007 for the un-weighted and weighted UniFrac distances, respectively, and ANOSIM showing R = 0.548 and P = 0.012, and R = 0.668 and P = 0.007 for the un-weighted and weighted UniFrac distances, respectively. These results indicated the significant differences in the microbiota composition between healthy and diseased shrimp (P < 0.05).

### Functional prediction of intestinal microbiota in healthy and diseased shrimp

**Fig 6:**
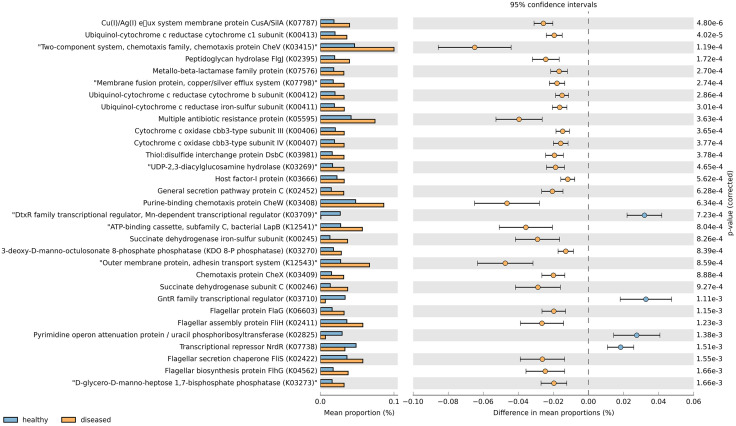
Functional feature prediction of intestinal microbiota from healthy (blue) and diseased (orange) shrimp. Fisher’s exact-test was employed to find significant differences of data between groups. P-value of the data was corrected using Newcombe-Wilson CI method and Benjamini-Hochberg FDR. Only the pathways with either significantly enhanced or suppressed by the occurrence of pale shrimp disease are shown. Data were generated based on 5 pooled intestinal samples from each group (n = 5).

Gut microbial functions predicted by PICRUSt2 based on the *16S rRNA* gene sequencing data revealed some dissimilarity between the healthy and diseased groups (Fig 6). Regarding KEGG level 2, pathways associated with cell motility, energy metabolism, and glycan biosynthesis and metabolism were enriched in the diseased shrimp, whereas that of biosynthesis of other secondary metabolites was decreased. In KEGG level 3, bacterial chemotaxis (K03415, K03408, and K03409), bacterial motility proteins (K03415, K02395, K03408, K03409, K06603, K02411, K02422, and K04562), flagellar assembly (K02395, K02411, and K02422), biofilm formation-*Vibrio cholera* (K03666 and K02452), two-component system (K07787, K00413, K03415, K07798, K00412, K00411, K00406, K00407, K03408, K00245, and K00246), oxidative phosphorylation (K00413, K00412, K00411, K00406, K00407, K00245, and K00246), secretion system (K03981, K02452, K12541, K12543, and K02411), and lipopolysaccharide biosynthesis (K03269, K03270, and K03273) were more abundant in the diseased group, while those of transcription factors (K03709, K03710, K02825, and K07738) were decreased. It is worth mentioning that functions associated with antibacterial resistance, including multiple antibiotic resistance protein (K05595) and metallo-β-lactamase family protein (K07576) were also significantly higher in the diseased shrimp. Nevertheless, bacterial functions related to carbohydrate, nucleotide and amino acid metabolism, and membrane transport were similar between the two groups.

## Discussion

Recently, PDD was identified as the etiology of pale shrimp disease in Thailand [1]. Coincidentally, PDD was also reported to cause “white muscle disease” in kuruma shrimp (*Marsupenaeus japonicus*) in aquaculture farms in Okinawa, Japan [31]. Due to the similar gross signs and the coinciding first occurrences at around the same time (mid-2022), it is possible that these two cases might be the same disease, but further investigation is needed to confirm this speculation. To the best of the authors’ knowledge, bacterial community structures in the gut of shrimp affected by pale shrimp disease have never been explored. In our study, the gut microbiota structure of pale shrimp from the outbreak pond was distinct from those of healthy shrimp as indicated by the composition and relative abundance of gut microbiota (Figs 1 and 2), shared and unique microbiota (Fig 3), α-diversity (Fig 4), and β-diversity indices (Fig 5). In one meta-analysis study, it was found that diseased shrimp usually contain more Gammaproteobacteria but less Alphaproteobacteria, Actinobacteria, Planctomycetes, and Verrucomicrobia compared to healthy shrimp [32]. Our results are consistent with this general pattern. At the genus level, *Photobacterium* and, to a lesser extent, *Vibrio*, predominated in 3 out of 5 intestinal samples from the diseased pond (i.e., the *Photobacterium*-*Vibrio* dominant type). As an opportunistic pathogen, it is perceivable that *Photobacterium* can be more abundant in the gut of diseased shrimp, such as those affected by AHPND [10,11] and WSSV [15], and shrimp exposed to lethal dose of nitrite [14], compared to healthy shrimp. Nevertheless, its proportion typically accounted for less than half of the total bacterial community. In our case, the percentage of *Photobacterium* in the *Photobacterium*-*Vibrio* dominant type of the diseased shrimp ranged from 54.63–70.53%, substantially higher than previously reported for other infectious diseases and environmental stress. Notably, although 7 ASVs of *Photobacterium* were shared between healthy and diseased shrimp, 3 unique *Photobacterium* ASVs, which were not present in the healthy shrimp, were found in the diseased shrimp (S1–S3 Tables). They might be highly pathogenic strain of *Photobacterium* and could possibly be responsible for causing pale shrimp disease in the affected pond. In addition, 10 unique ASVs of the *Vibrio* were detected in the disease group, which were much higher than those of the unique *Photobacterium* ASVs (S3 Table). However, oral inoculation of several *Vibrio* spp. isolated from the shrimp suffering pale shrimp disease failed to induce white muscle coloration. Thus, they were unlikely the main cause of pale shrimp disease in this case. This preliminary observation highlights the importance of *Photobacterium* in contributing to disease occurrence in the affected pond and supports findings from our recent research, which identified PDD as the etiological agent of pale shrimp disease [1]. Future research should focus on identifying factors associated with the pathogenesis of pale shrimp disease caused by *Photobacterium* through shotgun metagenomics with subsequent comprehensive bioinformatic and enzymatic/functional analyses. Such investigations would provide a more meaningful insights into the potential causative agents of the pale shrimp disease by specific strains of *Photobacterium*. It is worth mentioning that the microbiome analysis in this study was unable to accurately identify gut microbiota beyond the genus level. Further research is needed to determine the contribution of PDD within the *Photobacterium* genus in the intestines of diseased pale shrimp.

Interestingly, *Photobacterium* was not the prominent bacterium in the other 2 (out of 5) intestinal samples from the diseased pond. Instead, *Halarcobacter* and *Fusibacter* prevailed in this group. It should be noted that, during the initial stage of the outbreak, all individual shrimp are not uniformly affected but normally exhibit varying degrees of infection. Shrimp with a low abundance of *Photobacterium* are likely part of the population that has not yet been infected by PDD. The importance of *Halarcobacter* and *Fusibacter* in shrimp aquaculture has yet to be fully understood but might represent non-specific bacteria that colonize shrimp intestines at the early stage of the disease. *Fusibacter*, a sulfur-reducing bacterium that produces sulfide, was more abundant in the intestine of Pacific white shrimp suffering sulfide stress [16], and significantly enriched in the gut of black tiger shrimp (*Penaeus monodon*) from low-productive ponds, suggesting that it might be used as an indicator of productivity decay in shrimp pond [33]. Conversely, other studies revealed potential health benefits of *Fusibacter*. For instance, *Fusibacter* was identified as one of the key bacteria in the intestine of black tiger shrimp with higher body weight, occurring in greater abundance than in shrimp with lower body weight [34]. In addition, the antioxidant activity and immune response of Pacific white shrimp were found to be positively associated with *Fusibacter* in the intestine [35]. The role of *Halarcobacter* in shrimp health is even less clear; thus, additional study is necessary to gain a better understanding of its function. Regardless of the gut microbiota type (*Photobacterium*-*Vibrio* dominant or *Halarcobacter*-*Fusibacter* dominant), shrimp in the disease pond always had lower bacterial diversity than those from the healthy pond, with over 90% of their intestinal microbiota attributed to just 2 or 3 bacterial genera (Fig 1). Consequently, α-diversity indices (namely, observed feature, Shannon, and Simpson) of the gut microbiota from the outbreak pond were significantly lower than the healthy counterparts, and dominance of the gut microbiota from the outbreak pond was significantly higher than the healthy counterparts (Fig 4). Additionally, β-diversity showed significant difference between the two groups (Fig 5). The reduction in gut microbiome diversity (Fig 4) in the *Halacobacter–Fusibacter* dominant shrimp suggests that the onset of pale shrimp disease might be initiated by disruption of gut microbiota integrity due to unidentified causes which predisposing the shrimp to subsequent *Photobacterium* infection, and ultimately resulting in the manifestation of pale shrimp disease. It is commonly assumed that high biodiversity often (but not always) promotes the ecosystem stability [36–38], and loss of bacterial diversity (dysbiosis) is usually associated with increased susceptibility to diseases [19,39]. The lower bacterial diversity in the gut of diseased shrimp observed in this study was consistent with the findings of most previous research [7,9–11,40,41]. However, a few studies have reported either no significant difference [8,12,15,42] or even an opposite trend [21,32,43], implying that a decline in gut bacterial diversity alone is not a good indicator of health status [6,20]. Instead, an increase in the abundance of potential pathogens and alteration in microbial community structure have been proposed as more reliable indicators of dysbiosis [6].

The functional profile of the bacterial community in the diseased shrimp was different from the healthy shrimp (Fig 6). Predicted pathways that are directly or indirectly associated with the virulence of bacterial pathogens, including bacterial chemotaxis, bacterial motility, flagellar assembly, biofilm formation, secretion system, and two-component system [44–46] are more abundant in the diseased shrimp, suggesting that these functions may involve in pale shrimp disease pathogenesis. In addition, as pathways related to antibacterial resistance including metallo-β-lactamase family protein and multiple antibiotic resistance protein were significantly increased in the diseased group, it is likely that bacterial pathogens responsible for the disease outbreak in affected ponds might already developed antimicrobial resistance capability against β-lactam antibiotics as well as other drugs. The higher energy metabolism-related functions, especially oxidative phosphorylation, of the diseased shrimp compared to the normal shrimp, whereas those of nutrient metabolism profiles (i.e., carbohydrate, nucleotide, amino acid, and lipid metabolisms) showed no difference, imply that pathogenic bacteria may allocate more energy toward disease-causing activities, such as producing virulence factors or developing antimicrobial resistance. In addition, the disease might also impair normal physiological functions such as transcription process of the gut microbiota. Overall, these alterations in functional profile may contribute to the pale shrimp disease pathogenesis. However, it should be note that function profile prediction based on *16S rRNA* metagenomic sequencing could not confirm actual function of the pathogens. Thus, further investigation through metatranscriptome, metaproteome, and functional assay of pure isolate are highly recommended for validation of the obtained data.

By examining shrift in the gut microbiome of the Pacific white shrimp suffering pale shrimp disease, the obtained preliminary data revealed *Photobacterium* as the most likely etiological agent of the disease. However, the study had certain limitations, including a limited number of sample replicates and variable degrees of disease infection in individual samples. Future research should increase the number of replicates to obtain more robust data. Moreover, shotgun metagenomics, metatranscriptomics, metaproteomics, and functional assays would be valuable for validating the current findings, identifying factors associated with the pathogenesis of the highly virulent strain of *Photobacterium*, and elucidating the mechanisms underlying the onset and progression of pale shrimp disease-causing *Photobacterium*. Once these mechanisms are clarified, the highly virulent strain of *Photobacterium* can be routinely monitored for disease prevention and control, thereby helping reduce economic losses due to this pathogen.

## Conclusion

This study provides the first exploratory analysis of gut microbiota alterations in shrimp suffering pale shrimp disease. Although the number of biological replicates was limited in this case, the findings suggested that the disease was linked to marked shifts in the intestinal microbial composition, including enrichment of opportunistic taxa (*Photobacterium*), reduced microbial diversity, and enhanced metabolic pathways related to several virulent factors, antimicrobial resistance, and energy metabolism. These results highlight the pivotal role of gut microbiota in pale shrimp disease onset and progression, with *Photobacterium* appearing to play a significant role in the disease pathogenesis.

## Supporting information

S1 Fig Taxa bar plot of intestinal microbiota from healthy and diseased shrimp at the phylum level. The relative abundance of each intestinal microbiota is shown. Each bar represents the data of a pooled intestinal sample in each group (n = 5).
(TIFF)

S2 Fig Taxa bar plot of intestinal microbiota from healthy and diseased shrimp at the class level. The relative abundance of each intestinal microbiota is shown. Each bar represents the data of a pooled intestinal sample in each group (n = 5).
(TIFF)

S3 Fig Taxa bar plot of intestinal microbiota from healthy and diseased shrimp at the family level. The relative abundance of each intestinal microbiota is shown. Each bar represents the data of a pooled intestinal sample in each group (n = 5).
(TIFF)

S1 Table Shared intestinal microbiota between healthy and diseased shrimp. (XLSX)

S2 Table Unique intestinal microbiota in healthy shrimp. (XLSX)

S3 Table Unique intestinal microbiota in diseased shrimp. (XLSX)
