# Supplementary figures and images for: Exploring the gut microbiota of Pacific white shrimp (Litopenaeus vannamei) suffering pale shrimp disease
Source: PLoS One. 2025 Nov 11;20(11):e0336700. doi: 10.1371/journal.pone.0336700 (PMC12604756; doi:10.1371/journal.pone.0336700)

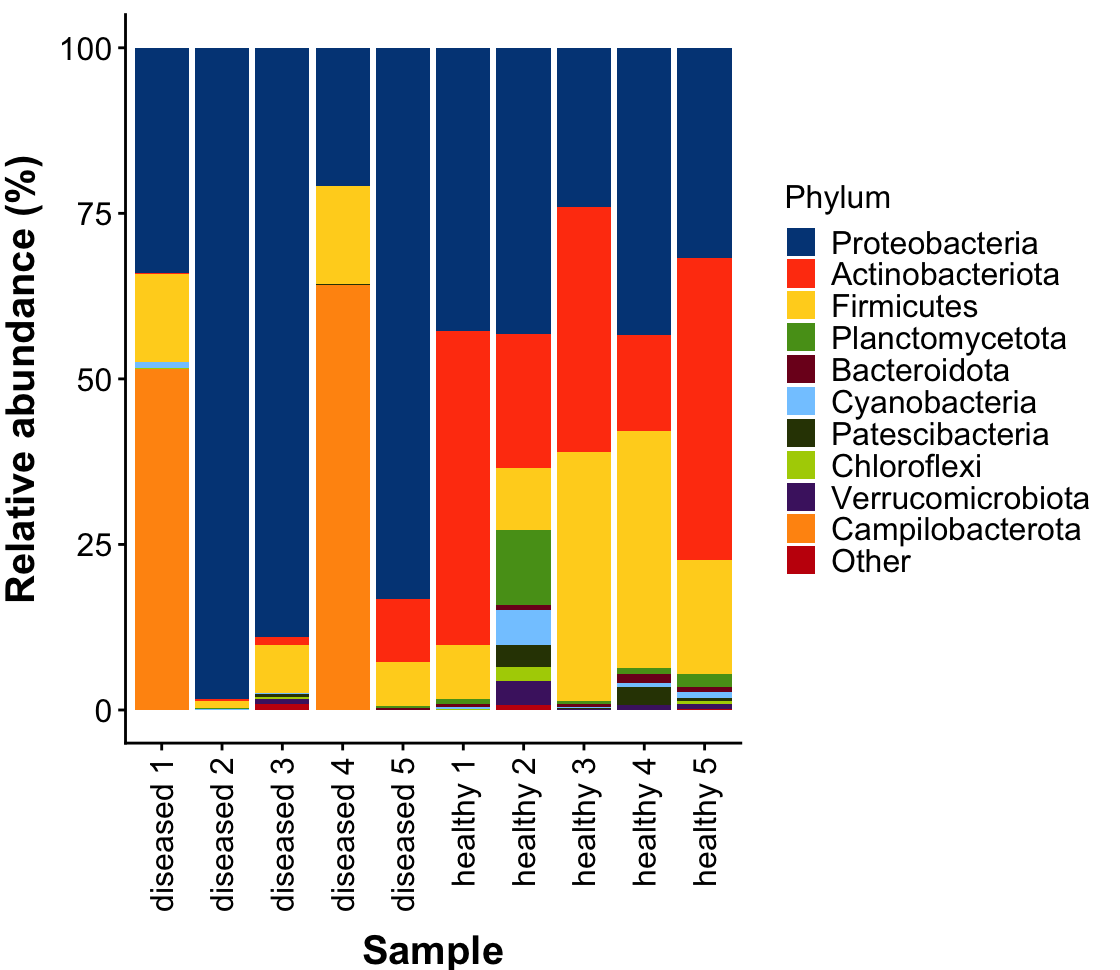

Supplement: S1 Fig — The relative abundance of each intestinal microbiota is shown. Each bar represents the data of a pooled intestinal sample in each group (n = 5). (TIFF) [file pone.0336700.s001.tiff]

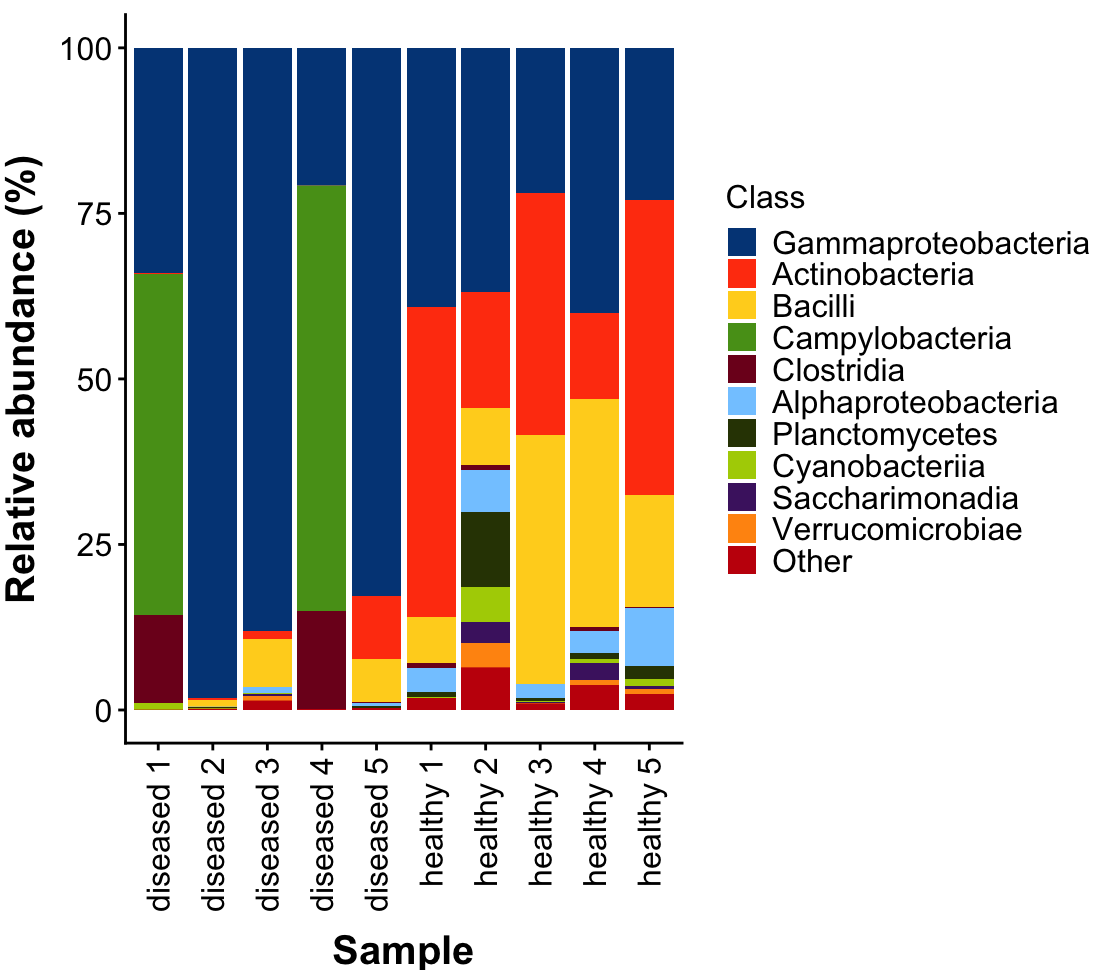

Supplement: S2 Fig — The relative abundance of each intestinal microbiota is shown. Each bar represents the data of a pooled intestinal sample in each group (n = 5). (TIFF) [file pone.0336700.s002.tiff]

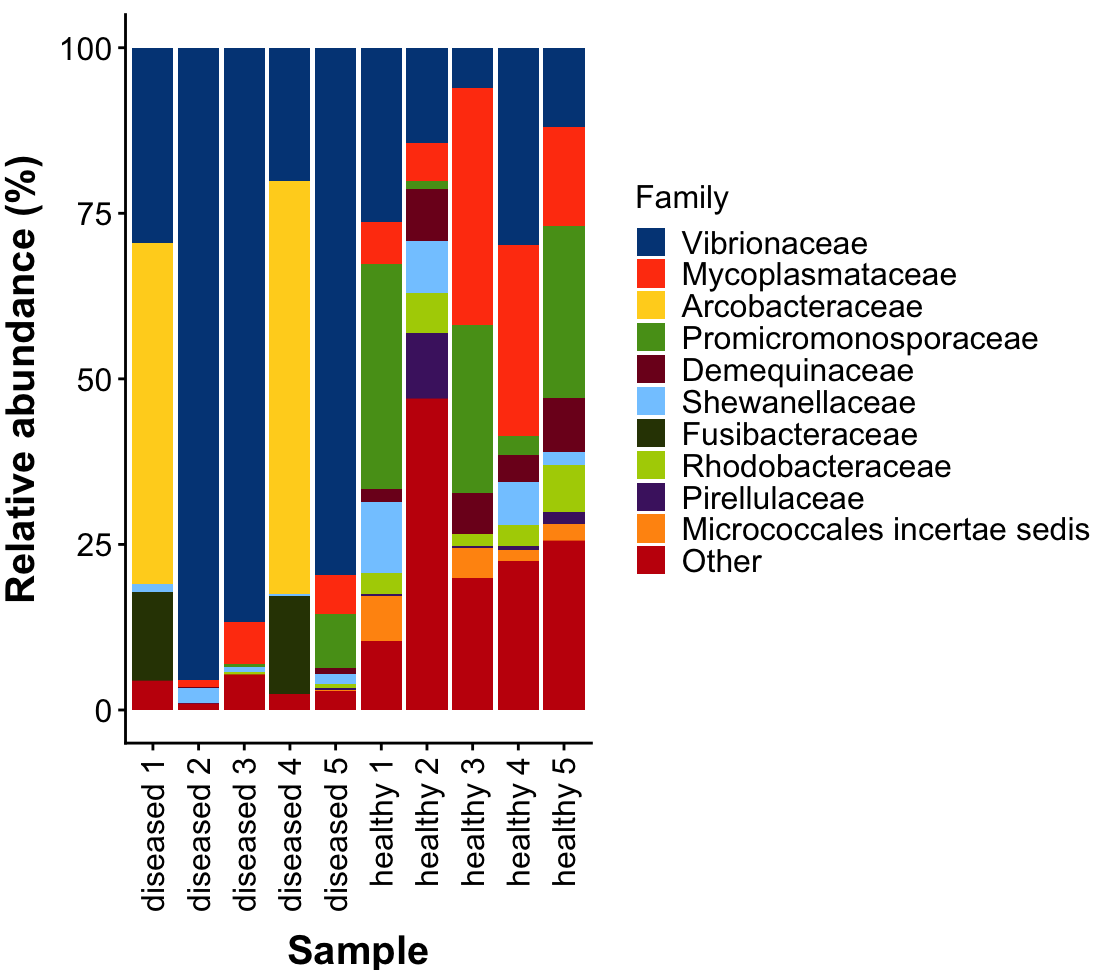

Supplement: S3 Fig — The relative abundance of each intestinal microbiota is shown. Each bar represents the data of a pooled intestinal sample in each group (n = 5). (TIFF) [file pone.0336700.s003.tiff]
